# Supplementary material for: Two halves are less than the whole: Evidence of a length bisection bias in fish (Poecilia reticulata)
Source: PLoS One. 2020 May 14;15(5):e0233157. doi: 10.1371/journal.pone.0233157 (PMC7224554; doi:10.1371/journal.pone.0233157)
Supplement: Attachment — Submitted filename: Response to reviewers.docx [file pone.0233157.s001.docx]

PONE-D-20-07425
Two halves are less that the whole: evidence of a length bisection bias in fish (Poecilia reticulata)
PLOS ONE

Dear Mrs. Santacà,

Thank you for submitting your manuscript to PLOS ONE. After careful consideration, we feel that it has merit but does not fully meet PLOS ONE’s publication criteria as it currently stands. Therefore, we invite you to submit a revised version of the manuscript that addresses the points raised during the review process.

So far I have had the manuscript reviewed by one expert on animal illusion perception. This reviewer supports the publication, but also raises a number of important revisions. I have read and agree with R1. In particular, I think you must more fully review animal literature on illusions, and hence reduce claims of this study being first evidence of a length bisection bias in an animal species. I also think there is a broader literature on spatial illusions like the Delboeuf illusion in animals that could be discussed. Please carefully address each point. I would also like that you make raw data available (see PLOS policies). If you can make all revisions in a careful way, I may proceed on the basis of the advice of the one expert reviewer.

Please include the following items when submitting your revised manuscript:

- A rebuttal letter that responds to each point raised by the academic editor and reviewer(s). This letter should be uploaded as separate file and labeled 'Response to Reviewers'.
- A marked-up copy of your manuscript that highlights changes made to the original version. This file should be uploaded as separate file and labeled 'Revised Manuscript with Track Changes'.
- An unmarked version of your revised paper without tracked changes. This file should be uploaded as separate file and labeled 'Manuscript'.

We look forward to receiving your revised manuscript.

Kind regards,

Adrian G Dyer, Ph.D.
Academic Editor
PLOS ONE

**## *Dear Prof. Dyer,***

***Thank you for your precious comments. We have now revised the manuscript according to all your suggestion and to the suggestions of the reviewer. In details, we have uploaded the data of the work, we corrected the title and we have broadly discussed the Delboeuf illusion. We have also reported a recent review that collects all the studies on the perception of visual illusions in fish. In particular, this year two different reviews have been published (Agrillo et al., 2020; Pecunioso et al., in press) that report all the previous literature both in fish (Agrillo et al., 2020) and in ‘lower’ vertebrates (Pecunioso et al., 2020); therefore, we have not included a broader discussion to other visual illusions.***

***On behalf of all authors,***

***Maria Santacà***

Journal Requirements:

When submitting your revision, we need you to address these additional requirements.

1. Please ensure that your manuscript meets PLOS ONE's style requirements, including those for file naming. The PLOS ONE style templates can be found at
   <https://journals.plos.org/plosone/s/file?id=wjVg/PLOSOne_formatting_sample_main_body.pdf> and
   <https://journals.plos.org/plosone/s/file?id=ba62/PLOSOne_formatting_sample_title_authors_affiliations.pdf>

**## *We have now modified and renamed the files to meet PLOS ONE’s style requirements.***

1. Please update the word "that" in the title to "than" for grammatical correctness.

**## *We apologize for the mistake. We have now corrected the title (line 4).***

2. We note that you have indicated that data from this study are available upon request. PLOS only allows data to be available upon request if there are legal or ethical restrictions on sharing data publicly. For more information on unacceptable data access restrictions, please see <http://journals.plos.org/plosone/s/data-availability#loc-unacceptable-data-access-restrictions>.

In your revised cover letter, please address the following prompts:

a) If there are ethical or legal restrictions on sharing a de-identified data set, please explain them in detail (e.g., data contain potentially sensitive information, data are owned by a third-party organization, etc.) and who has imposed them (e.g., an ethics committee). Please also provide contact information for a data access committee, ethics committee, or other institutional body to which data requests may be sent.

b) If there are no restrictions, please upload the minimal anonymized data set necessary to replicate your study findings as either Supporting Information files or to a stable, public repository and provide us with the relevant URLs, DOIs, or accession numbers. For a list of acceptable repositories, please see <http://journals.plos.org/plosone/s/data-availability#loc-recommended-repositories>.

We will update your Data Availability statement on your behalf to reflect the information you provide.

**## *As suggested also by the reviewer, we have now added the data of the study as supplementary materials (see file S1 Table)***.

3. Your ethics statement must appear in the Methods section of your manuscript. If your ethics statement is written in any section besides the Methods, please move it to the Methods section and delete it from any other section. Please also ensure that your ethics statement is included in your manuscript, as the ethics section of your online submission will not be published alongside your manuscript.

**## *We have moved the ethics statement in the Methods section (lines 109-113).***

Reviewers' comments:

Reviewer's Responses to Questions

**Comments to the Author**

1. Is the manuscript technically sound, and do the data support the conclusions?

The manuscript must describe a technically sound piece of scientific research with data that supports the conclusions. Experiments must have been conducted rigorously, with appropriate controls, replication, and sample sizes. The conclusions must be drawn appropriately based on the data presented.

Reviewer #1: Yes

2. Has the statistical analysis been performed appropriately and rigorously?

Reviewer #1: Yes

3. Have the authors made all data underlying the findings in their manuscript fully available?

The [PLOS Data policy](http://www.plosone.org/static/policies.action#sharing) requires authors to make all data underlying the findings described in their manuscript fully available without restriction, with rare exception (please refer to the Data Availability Statement in the manuscript PDF file). The data should be provided as part of the manuscript or its supporting information, or deposited to a public repository. For example, in addition to summary statistics, the data points behind means, medians and variance measures should be available. If there are restrictions on publicly sharing data—e.g. participant privacy or use of data from a third party—those must be specified.

Reviewer #1: No

4. Is the manuscript presented in an intelligible fashion and written in standard English?

PLOS ONE does not copyedit accepted manuscripts, so the language in submitted articles must be clear, correct, and unambiguous. Any typographical or grammatical errors should be corrected at revision, so please note any specific errors here.

Reviewer #1: Yes

5. Review Comments to the Author

Please use the space provided to explain your answers to the questions above. You may also include additional comments for the author, including concerns about dual publication, research ethics, or publication ethics. (Please upload your review as an attachment if it exceeds 20,000 characters)

Reviewer #1: The authors studied the HV visual illusion in guppies. They found that guppies were susceptible to the illusion when a line bisected the horizontal line - but this was not the case with 'L-shaped' stimuli. I would recommend this manuscript for publication with some minor revisions:

1. The authors state that there are some restrictions on data availability. Is this necessary? Why can the data not be publicly accessible?

**## *We have now added a supplementary file with the data of each tested subject (see S1 Table).***

2. The title has an grammatical error. It currently reads: "Two halves are less THAT the whole: evidence of a length bisection bias in fish (Poecilia reticulata)". I think the authors meant to write: "Two halves are less THAN the whole: evidence of a length bisection bias in fish (Poecilia reticulata)"

**## *We do apologize for the mistake. We have now corrected the title changing “that” in “than” (line 4).***

3. Line 15 - "explores" should be "explore".

**## *We changed it accordingly (line 18).***

4. I am confused by the claim the authors make multiple times (e.g. line 25-26 "... providing the first evidence of a length bisection bias in an animal species." I thought that this has been shown in non-human primates and hens? If this is the case, the lines and the other claims like it throughout the manuscript should be removed.

**## *The previous studies on monkeys and hens did not dissociate the exact role of anisotropy of the perceived space in the vertical and horizontal space – due to the shape of animals’ visual field - and the length bisection bias.***

***Here we found evidence that a bisected line appears to be shorter in a guppy brain. On the contrary guppies do not show a length misperception as a function of the vertical vs. horizontal presentation. In this sense we claimed we found the ‘very first evidence’ of length bisection bias in animals. That said, we acknowledge we cannot exclude that length bisection bias might have played a role in primates and hens, therefore in the revised version we wrote we provided the first evidence of the length bisection bias in a fish species (lines 26, 106, 281).***

5. Line 144 - "breed" should be "bred".

**## *We changed it accordingly (line 115).***

6. Line 227 - "subjects" should be changed to "subject" as the authors state that only one guppy took the 120 trials instead of the 24 trials of the other 11 guppies.

**## *The reviewer is right. We changed it accordingly (line 236).***

7. Lines 258-260 - The first sentence of the discussion is messy and should be revised. The second part of the sentence does not make sense: "... that the results of which experiment have the capacity to shed light on the evolution of perceptual mechanisms underlying size estimation of vertebrates."

**## *As suggested, we revised and simplified the first sentence of the discussion (lines 267-269).***

8. Line 258 - "is susceptible" should be changed to "are susceptible" for grammar purposes.

**## *We changed it accordingly (line 267).***

9. Line 270 - Change "human" to "humans".

**## *We changed it accordingly (line 278).***

10. Line 270 - Change "primate" to "primates".

**## *We changed it accordingly (line 278).***

11. 270 - delete the word "so".

**## *We removed ‘so’ as suggested.***

12. Lines 280-284 - This is a very long sentence and should be broken up and made clearer. Currently it is confusing. The Delboeuf illusion should also be explained better for readers to understand how/why humans perceive it in a certain way and how/why guppies perceive it differently.

**## *We have now rewritten this part to make it clearer. In particular, we defined both the Muller-Lyer illusion and the Delboeuf illusion; we have also reported how these illusions work in humans and how guppies showed to perceive it (lines 288-311).***

6. PLOS authors have the option to publish the peer review history of their article ([what does this mean?](https://journals.plos.org/plosone/s/editorial-and-peer-review-process#loc-peer-review-history)). If published, this will include your full peer review and any attached files.


If you choose “no”, your identity will remain anonymous but your review may still be made public.


**Do you want your identity to be public for this peer review?** For information about this choice, including consent withdrawal, please see our [Privacy Policy](https://www.plos.org/privacy-policy).

Reviewer #1: No

While revising your submission, please upload your figure files to the Preflight Analysis and Conversion Engine (PACE) digital diagnostic tool, <https://pacev2.apexcovantage.com/>. PACE helps ensure that figures meet PLOS requirements. To use PACE, you must first register as a user. Registration is free. Then, login and navigate to the UPLOAD tab, where you will find detailed instructions on how to use the tool. If you encounter any issues or have any questions when using PACE, please email us at [figures@plos.org](mailto:figures@plos.org). Please note that Supporting Information files do not need this step.

**## *We uploaded all the figures to the Pace digital diagnostic tool and all were approved.***
