# Supplementary material for: Two halves are less than the whole: Evidence of a length bisection bias in fish (Poecilia reticulata)
Source: PLoS One. 2020 May 14;15(5):e0233157. doi: 10.1371/journal.pone.0233157 (PMC7224554; doi:10.1371/journal.pone.0233157)
Supplement: Attachment — Submitted filename: Response to reviewers.docx [file pone.0233157.s002.docx]

PONE-D-20-07425R1
Two halves are less than the whole: evidence of a length bisection bias in fish (Poecilia reticulata)
PLOS ONE

Dear Mrs. Santacà,

Thank you for submitting your manuscript to PLOS ONE. After careful consideration, we feel that it has merit but does not fully meet PLOS ONE’s publication criteria as it currently stands. Therefore, we invite you to submit a revised version of the manuscript that addresses the points raised during the review process.

I have carefully read the revised manuscript which I find very interesting. I have however two suggestions that I think would improve the manuscript for readers.

AGD1 Given the size of the Sup Table S1 containing the data requested by reviewer 1 (and myself to be compliant with PLOS1 policies) is not very large, I would like to suggest you just add this table to the main mainscript so that all information is at hand for readers of the manuscript. Else if you want to retain this information only as sup file I think it would be good to label that file so that when it is downloaded it is clear what the information relates to. But as stated above, if the information is placed in the main manuscript for readers and clearly labled it might be best? I will leave this decision to you.

AGD2 Regarding your revised text ["In the latter case, guppies showed not to overestimate the size of a circle presented in a small circumference. On the contrary, they demonstrated to underestimate the size of such circle and to overestimate the size of the same circle but presented in a large circumference suggesting a reverse perception of the Delboeuf illusion compared to humans [19]."]. I found this section of text not very clear. Do you mean [guppies showed evidence of not overestimating size.....]? I found the logic of these 2 sentences difficult to understand as written; can you please re write and cross check with lab members so we are confident that the general readers of PLOS will easily understand you meaning?

If you can attend to these two points I am confident I will be able to accept the manuscript.

We would appreciate receiving your revised manuscript by Jun 12 2020 11:59PM. When you are ready to submit your revision, log on to <https://www.editorialmanager.com/pone/> and select the 'Submissions Needing Revision' folder to locate your manuscript file.

If you would like to make changes to your financial disclosure, please include your updated statement in your cover letter.

To enhance the reproducibility of your results, we recommend that if applicable you deposit your laboratory protocols in [protocols.io](http://protocols.io/), where a protocol can be assigned its own identifier (DOI) such that it can be cited independently in the future. For instructions see: <http://journals.plos.org/plosone/s/submission-guidelines#loc-laboratory-protocols>

Please include the following items when submitting your revised manuscript:

- A rebuttal letter that responds to each point raised by the academic editor and reviewer(s). This letter should be uploaded as separate file and labeled 'Response to Reviewers'.
- A marked-up copy of your manuscript that highlights changes made to the original version. This file should be uploaded as separate file and labeled 'Revised Manuscript with Track Changes'.
- An unmarked version of your revised paper without tracked changes. This file should be uploaded as separate file and labeled 'Manuscript'.

Please note while forming your response, if your article is accepted, you may have the opportunity to make the peer review history publicly available. The record will include editor decision letters (with reviews) and your responses to reviewer comments. If eligible, we will contact you to opt in or out.

We look forward to receiving your revised manuscript.

Kind regards,

Adrian G Dyer, Ph.D.
Academic Editor
PLOS ONE

**## *Dear Prof. Dyer,***

***Thank you for your precious comments. We have revised the manuscript according to both your suggestions. We have now inserted the supplementary table in the manuscript and we simplified the part of the Delboeuf illusion perception in guppies to make it clearer (lines 298-299).***

***On behalf of all authors,***

***Maria Santacà***
